# VAMP-Associated Protein B (VAPB) Promotes Breast Tumor Growth by Modulation of Akt Activity

**DOI:** 10.1371/journal.pone.0046281

**Published:** 2012-10-01

**Authors:** Meghana Rao, Wenqiang Song, Aixiang Jiang, Yu Shyr, Sima Lev, David Greenstein, Dana Brantley-Sieders, Jin Chen

**Affiliations:** 1 Department of Cancer Biology, Vanderbilt University School of Medicine, Nashville, Tennessee, United States of America; 2 Department of Medicine, Vanderbilt University School of Medicine, Nashville, Tennessee, United States of America; 3 Department of Cellular and Developmental Biology, Vanderbilt University School of Medicine, Nashville, Tennessee, United States of America; 4 Department of Biostatistics, Vanderbilt University School of Medicine, Nashville, Tennessee, United States of America; 5 Vanderbilt-Ingram Cancer Center, Vanderbilt University School of Medicine, Nashville, Tennessee, United States of America; 6 Veterans Affairs Medical Center, Tennessee Valley Healthcare System, Nashville, Tennessee, United States of America; 7 Department of Molecular Cell Biology, Weizmann Institute of Science, Rehovot, Israel; 8 Department of Genetics, Cell Biology and Development, University of Minnesota, Minneapolis, Minnesota, United States of America; Wake Forest University, United States of America

## Abstract

VAPB (VAMP- associated protein B) is an ER protein that regulates multiple biological functions. Although aberrant expression of VAPB is associated with breast cancer, its function in tumor cells is poorly understood. In this report, we provide evidence that VAPB regulates breast tumor cell proliferation and AKT activation. VAPB protein expression is elevated in primary and metastatic tumor specimens, and VAPB mRNA expression levels correlated negatively with patient survival in two large breast tumor datasets. Overexpression of VAPB in mammary epithelial cells increased cell growth, whereas VAPB knockdown in tumor cells inhibited cell proliferation *in vitro* and suppressed tumor growth in orthotopic mammary gland allografts. The growth regulation of mammary tumor cells controlled by VAPB appears to be mediated, at least in part, by modulation of AKT activity. Overexpression of VAPB in MCF10A-HER2 cells enhances phosphorylation of AKT. In contrast, knockdown of VAPB in MMTV-Neu tumor cells inhibited pAKT levels. Pharmacological inhibition of AKT significantly reduced three-dimensional spheroid growth induced by VAPB. Collectively, the genetic, functional and mechanistic analyses suggest a role of VAPB in tumor promotion in human breast cancer.

## Introduction

Vesicle associated membrane protein associated protein B (VAPB) is a highly conserved type II integral membrane protein that belongs to the VAP protein family [1], [2] and primarily localizes to the endoplasmic reticulum (ER) and cis-Golgi [3], [4]. Studies of VAP-interacting proteins in yeast and in higher organisms implicate VAP proteins in a diverse array of cellular processes. VAP proteins function in the regulation of neurotransmitter release, vesicle trafficking, lipid binding and transfer proteins, maintainance of ER/golgi architecture and the unfolded protein response (UPR) [5], [6], [7], [8]. Recent studies in C. elegans and Drosophila discovered that the MSP domain of VAPB can be cleaved, secreted, and act as a ligand for Eph receptor tyrosine kinases [9]. A single missense mutation within the human VAPB gene is associated in a familial form of atypical amyotrophic lateral sclerosis (ALS) [10], [11], [12], triggering a renewed interest in the VAPB protein and its cellular function in human pathologies.

In addition to familial ALS, VAPB expression has been observed with cancer. A genome-wide microarray analysis of 50 human breast cancer cell lines and 145 clinical specimens revealed that VAPB is often amplified or overexpressed in breast cancer [13], [14]. As a potential Eph receptor ligand, the MSP domain of the VAPB protein could affect tumor growth and invasion through modulation of Eph receptor activity, which is commonly dysregulated in cancer [15], [16]. Furthermore, as VAPB also functions in protein secretion and vesicle trafficking, tumor cells may rely on this pathway for receptor localization and growth factor secretion in order to sustain growth [17]. Despite strong indications of the consequences of VAPB expression in cancer, a direct role of VAPB in tumor growth has not been investigated.

In this report, we studied the role of VAPB in breast cancer. We analyzed the expression of VAPB in both a breast cancer tissue microarray and two published large mRNA expression datasets and correlated VAPB expression with clinical outcomes. To determine the causal role of VAPB in cancer, we overexpressed VAPB in mammary epithelial cells or stably knocked down VAPB in tumor cells. Cell proliferation, apoptosis, spherical growth in 3D culture, and tumor growth in vivo were analyzed. Finally, we identified molecular mechanisms by which VAPB regulates tumor cell proliferation.

**Figure 1:**
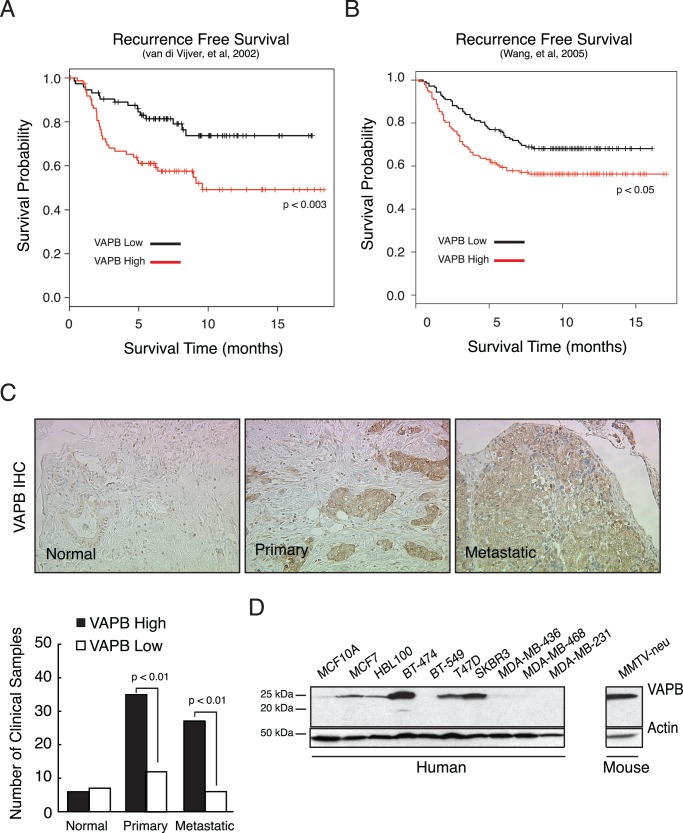
Analysis of VAPB expression in human breast cancer. VAPB expression levels were analyzed in two published breast cancer microarray datasets [19]–[20]. Correlation of VAPB expression with clinical outcome was analyzed by Kaplan-Meier survival curve using the van de Vijver dataset (A) [19] and the Wang dataset (B) [20] (C) VAPB protein expression was analyzed in human invasive ductal carcinoma samples on commercial tissue microarrays by immunohistochemistry using a previously validated anti-VAPB antibody, as described in Materials and Methods (D) Western analysis of VAPB protein in human breast cancer cell lines.

## Materials and Methods

### Antibodies, Reagents and Plasmids

Antibodies against the following proteins were used: anti-VAPB (K-16), anti-VAPA (K-14), anti-actin (I-19), anti-β-tubulin (Santa Cruz Biotechnologies), and anti-PCNA (NeoMarkers), anti-Myc, anti-cleaved casepase 3, anti-pThr308AKT, anti-pSer473AKT, anti-pERK, anti-AKT, anti-ERK (Cell Signaling Technologies). Lentiviral control and VAPB shRNA plasmids KD# 1 (5′-GCACACACAAATATAGCATAA-3′) and KD# 2 (5′-CGGAAGACCTTATGGATTCAA-3′) and VAPB cDNA were obtained from OpenBiosystems. Growth factor-reduced Matrigel and TO-PRO3 was purchased from BD Biosciences and Invitrogen, respectively. The AKT 1/2 inhibitor 5J8/0360263-1 was produced by the Vanderbilt University Department of Chemistry as described previously [18].

**Figure 2:**
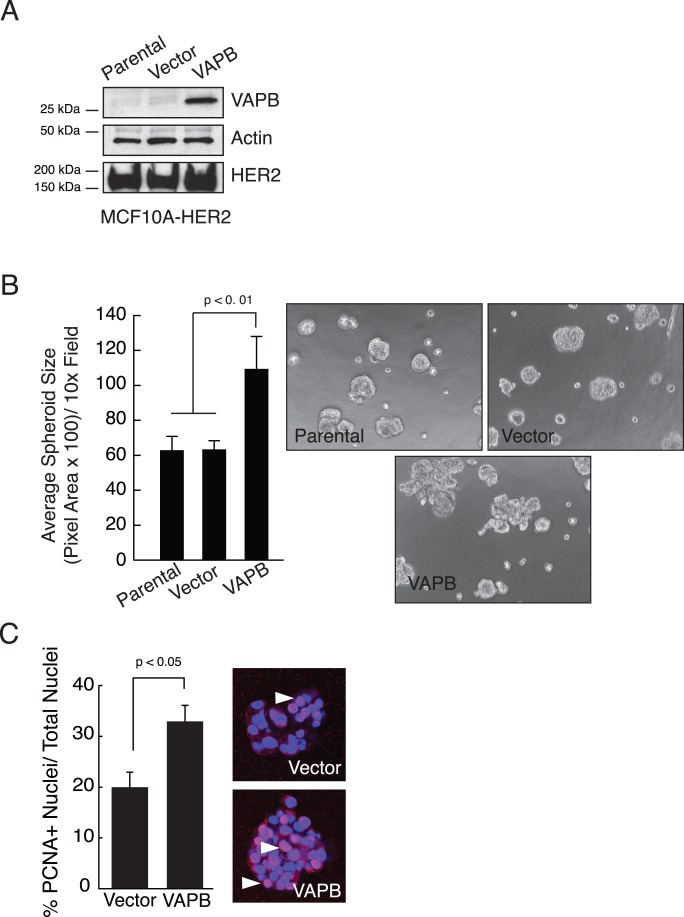
VAPB expression enhances spheroid size and proliferation in mammary epithelial cells. (A) VAPB was stably expressed in MCF10A-HER2 cells via retroviral transduction, as judged by western blot analysis. (B) MCF10A-HER2-VAPB spheroids were cultured in three-dimensional Matrigel. Spheroid size was quantified and presented as average pixel area per spheroid (p<0.01, ANOVA). (C) Cell proliferation in spheroid culture was determined by immunofluorescence using an anti-PCNA antibody (red). Cell nuclei were stained blue by TO-PRO-3 nuclear stain. PCNA-positive nuclei were quantified from an average of 15 random spheroids for each of two experiments (p<0.05, t test). Arrows: PCNA positive cells.

### Human mRNA Expression Profiling and Tissue Microarray Protein Analyses

Analysis of VAPB expression in human breast cancer datasets [19], [20] was performed in collaboration with the Vanderbilt-Ingram Cancer Center’s Biostatistics Core Resource. VAPB “high” or “low” expression was defined as top or bottom quartiles of tumors expressing VAPB. Expression levels were analyzed in relation to overall and/or recurrence-free survival using Log Rank and Cox analyses. Statistical analyses were performed using Software: R2.12.1. The survival curves from Kaplan-Meier was created and plotted by the function “survfit” under R package “survival.” P values shown on KM plots were calculated based on log rank test between two survival curves of high or low expression groups.

**Figure 3:**
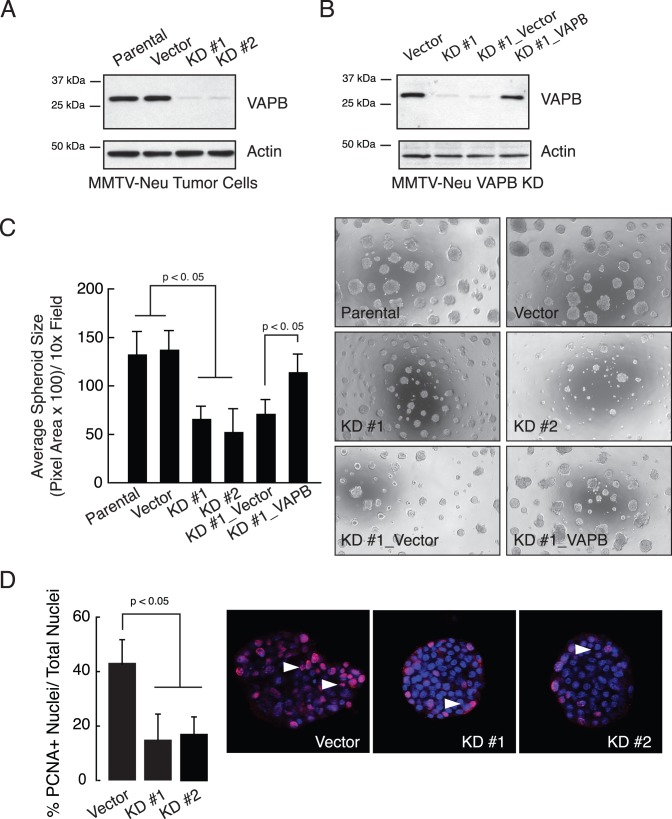
VAPB knockdown impairs mammary tumor spheroid growth and cell proliferation. (A) VAPB expression was silenced in MMTV-Neu tumor cells by two independent lentiviral-mediated shRNAs, as judged by western blot analysis. (B) VAPB knockdown (KD #1) cells were rescued by re-expressing a full-length human VAPB cDNA via retroviral transduction. (C) MMTV-Neu spheroids were cultured in three-dimensional Matrigel. Spheroid size was quantified and presented as average pixel area per spheroid (p<0.01, ANOVA). (D) MMTV-Neu cell proliferation in spheroid culture was determined by immunofluorescence using an anti-PCNA antibody (red). PCNA-positive nuclei were quantified from an average of 15 random spheroids for each of two experiments (p<0.05, t test). Arrows: PCNA positive cells.

Breast tumor spectrum Tissue Microarrays (TMAs; BR480) were purchased from US Biomax, Inc. (Rockville, MD). Immunohistochemical staining for VAPB was performed as described previously [15], [21] using a previously validated rabbit anti-VAPB antibody [22].Briefly, tumor sections were re-hydrated and subjected to thermal antigen retrieval in citrate buffer (2 mM citric acid, 10 mM sodium citrate, pH 6.0) using a PickCell Laboratories 2100 Retriever as per manufacturer’s instructions. Endogenous peroxidases were quenched by incubation in 3% H_2_O_2_ solution for 30 minutes. After blocking, sections were incubated with anti-VAPB (1∶25, produced in the Lev lab) overnight at 4°C, followed by a biotinylated goat anti-rabbit secondary antibody (1∶200, BD Pharmingen, San Diego, CA), an avidin-peroxidase reagent (Life Technologies/Molecular Probes, Carlsbad, CA), and stained with liquid 3,3′-diaminobenzidine tetrahydrochloride (DAB) substrate kit (Life Technologies/Zymed Laboratories). Relative expression in TMAs was scored using a continuous scale as [22] follows: 0=0−10% positive tumor epithelium, 1=10−25% positive tumor epithelium, 2=25−50% positive tumor epithelium, and 3= >50% positive tumor epithelium/core. Differential expression between tissue samples was quantified and analyzed statistically (Chi square analysis).

**Figure 4:**
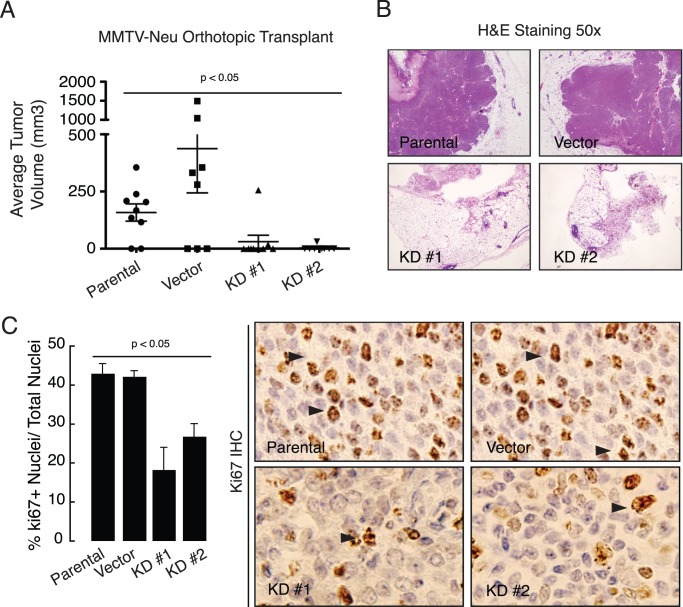
VAPB is required for tumor growth *in vivo*. (A) One million of MMTV-Neu control or VAPB knockdown cells were injected into cleared mammary gland fat pads of 3-week old FVB recipient female mice (n=8−10/experimental condition). Tumors were harvested 5 weeks after transplantation. Tumor size was measured by a caliper and tumor volumes were calculated. (p<0.05, ANOVA). (B) Tumors were processed, sectioned and stained with H&E (B) or Ki67, a proliferation marker (C). Proliferating tumor cells were quantified by enumeration of Ki67+ nuclei and presented as a percentage of Ki67+ nuclei/total nuclei. VAPB knockdown cells showed a significant decrease in proliferation (p<0.05, ANOVA). Arrows: Ki67 positive cells.

### Cell Culture and Generation of Stable VAPB Knockdown or Overexpression Cell Populations

MMTV-Neu tumor cells were isolated as previously described [23] and grown in DMEM/F-12 (Invitrogen) supplemented with 10% Fetal Bovine Serum (FBS) (Hyclone), 5 ng/mL estrogen (Sigma-Aldrich), 5 ng/mL progesterone (Sigma-Aldrich), 5 ng/mL EGF (PreproTech), 5 µg/mL insulin (Sigma-Aldrich) with penicillin/streptomycin. For generation of stable VAPB knockdown cells, Vector-only (pLKO.1) or VAPB shRNA lentivirus was produced by co-transfecting HEK-293T cells with pLKO.1 or pLKO.1-shRNAs, along with packaging plasmids psPAX2 and pMD.2G, using Lipofectamine. Viral supernatants were collected 48 and 72 hours post transfection and used to infect MMTV-Neu tumor cells. MMTV-Neu cells were selected for plasmid inclusion by culturing cells in growth media containing 5 µg/mL of puromycin (Sigma-Aldrich). For re-expression of VAPB, VAPB cDNA (open reading frame) was subcloned into pCLSXN retroviral vector that contains a Neomycin resistance gene, allowing for G418 (300 µg/mL for 6 days) selection in puromycin-resistant populations. Viral supernatant was collected as described above from HEK 293T cells that were co-transfected with pCLSXN-Vector (10 µg) only or pCLSXN-VAPB (10 µg) and pCLAmpho plasmids (10 µg) using Lipofectamine 2000.

**Figure 5:**
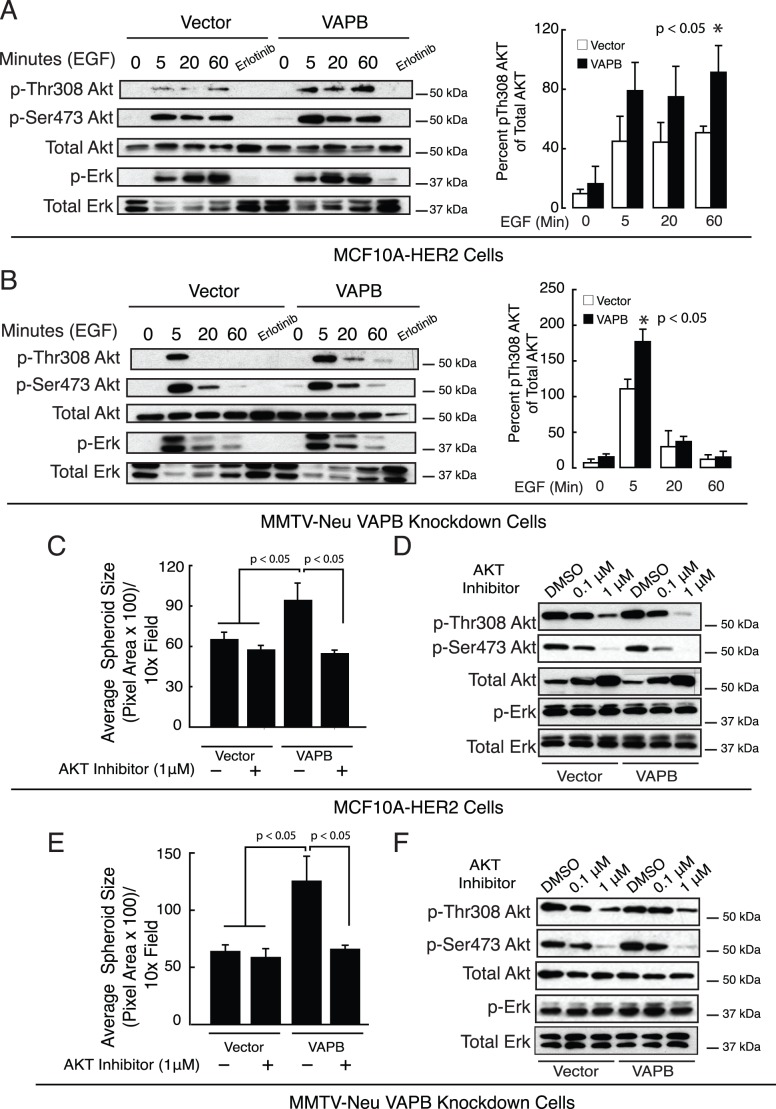
VAPB-dependent cell growth is mediated through AKT activity. (A) MCF-10A cells expressing VAPB or carrying control vector were serum starved and stimulated with 20 ng/mL EGF at the indicated time points. Phospho-AKT levels in MCF10A-HER2 cells were measured by western blot analysis. (B) VAPB was knocked down in MMTV-Neu cells (KD#1) and re-expressed via retroviral transduction (KD#1_VAPB). Phospho-AKT levels were measured by western blot analysis Representative blots from 3 independent experiments are shown. (C–E) Pharmacologic AKT inhibition significantly impaired VAPB-mediated spheroid growth in MCF10A-Her2 cells (C) and MMTV-Neu VAPB knockdown cells rescued with VAPB reexpression (E) (p<0.05 ANOVA). Inhibition of AKT activity was confirmed by western blot analysis for phospho-AKT in MCF10A-HER2 cells (D) and MMTV-Neu knockdown cells (F).

MCF10A cells were obtained from American Type Culture Collection (ATCC) and cultured as previously described [24]. MCF10A-HER2 cells were generated via retroviral transduction of the HER2 proto-oncogene [25]. Overexpression of VAPB in MCF10A-HER2 cells was achieved by infecting cells with pCLXSN-Vector or -VAPB retrovirus and selected in media containing 300 µg/mL G418 for 6 days.

### Analysis of 3-dimensional Spheroid Culture

MMTV-Neu tumor cells or MCF10A-HER2 cells were plated on a solidified layer of Matrigel (2 mm thick) using an 8-well, glass chamber slide (Thermo Scientific) as previously described [15], [24]. Fresh MMTV-Neu growth media or MCF10A-HER2 Assay Media [24] was replaced every 48 hours. Cultures were maintained for 9 days prior to photo-documentation. Digital images were taken in 4 random fields/well (3–4 spheroids/field). Total spheroid area was calculated using NIH Image J software. For spheroid proliferation analysis, cultures were fixed at day 5 in a 1∶1 methanol: acetone solution for 10 minutes at −20°C and co-stained with anti-PCNA (1∶1000) and nuclear stain TO-PRO3 (1∶2000) as previously described [24]. Confocal images of 15 spheroids per experiment were taken at random and percentage of PCNA-positive nuclei was quantified. For AKT inhibitor-treated spheroid cultures, cells were allowed to grow for 48 hours, followed by daily AKT-inhibitor or vehicle control treatment starting on day3. For each analysis described above, at least two to three independent experiments were performed.

### *In vivo* Tumor Studies

All animals were housed under pathogen-free conditions, and experiments were performed in accordance with AAALAC guidelines and with Vanderbilt University Institutional Animal Care and Use Committee approval. FVB female recipient animals were cleared of endogenous epithelium as described previously [26] and one million MMTV-Neu tumor cells were injected orthotopically. A total of 8–10 animals per group were analyzed in 2 independent experiments. Resulting tumors were harvested 4–5 weeks after injection for analysis of tumor volume (volume=length × width^2^ × 0.52). Harvested tumors were further processed for H&E staining and immunohistochemistry analysis of proliferation (Ki67) or apoptosis (cleaved caspase-3). Proliferating tumor cells were quantified by enumerating Ki67 positive nuclei in 4 random fields per tumor and presented as a percentage of Ki67+nuclei/total nuclei. A total of 8–10 tumors were quantified in two independent experiments. Statistical significance was assessed by single factor ANOVA.

### Immunoblotting

For immunoblot analysis of VAPB knockdown or overexpression, cells were washed with PBS and lysed in Radioimmunoprecipitation Assay (RIPA) buffer (50 mM Tris pH 7.4, 150 mM NaCl, 1% NP-40, 0.5% deoxycholic acid, 0.1% SDS) supplemented with protease and phosphatase inhibitor tablets (Roche). Approximately 30–50 ug of total cell lysate was separated by SDS-PAGE and probed with anti-VAPB (1∶1000) overnight in 5% nonfat dry milk/TBS-Tween (0.05%) and detected with anti-goat HRP (1∶1000). For analysis of pAKT levels in VAPB expressing cells, cells were serum starved overnight and stimulated with DMEM/F-12 with 20 ng/mL of EGF for 20 minutes. For some experiments, an AKT inhibitor was added under normal growth conditions for 3 hours prior to cell lysis. Data are a representation of three independent experiments.

### Statistical Analysis

Results are presented as mean values±standard error. *P*-values are given in the figure legends, and values of *P*<0.05 were considered to be statistically significant. Statistical analyses were performed by single factor ANOVA and unpaired, two-tailed Student *t*-test using PRISM software (GraphPad Sofware, La Jolla, CA, USA). For TMAs, statistical significance of VAPB expression between patient samples was determined by Chi-Square analysis.

## Results

### VAPB is Overexpressed in Breast Cancer and Negatively Correlates with Patient Survival

Several studies reported an increase in VAPB copy number or expression in breast cancer [13], [14], [27], [28], [29]. To determine the impact of VAPB expression on clinical outcome in human breast cancer, we analyzed VAPB expression in a published human breast cancer microarray dataset from a panel of 295 patient samples [19]. Kaplan-Meier analysis revealed that high levels of *VAPB* mRNA were significantly associated with poorer recurrence free survival (Figure 1A) than in patients with low *VAPB* mRNA expression. Similar results were obtained from a second, independent patient data set (n=286) (Figure 1B) [20]. To assess VAPB protein expression in human breast cancer, we performed immunohistochemistry in breast cancer tissue microarrays (TMA, n=84) with a previously validated anti-VAPB antibody [22]. As shown in Figure 1C, we observed a significant increase of VAPB expression in invasive ductal carcinoma and lymph node metastasis samples, compared to normal tissue (p<0.01, Chi-square test). Taken together these data highlight the clinical relevance of high VAPB expression in human breast cancer.

### VAPB Promotes Tumor Spheroid Growth by Enhancing Tumor Cell Proliferation

Because the VAPB allele is often amplified in human breast cancer [13], [14], including cancers that have HER2-amplification [27],we sought to determine if elevated VAPB expression enhances tumor cell growth. MCF10A-HER2 cells, an immortalized mammary epithelial cell line expressing the HER2 oncogene, were transduced to express human VAPB protein (Figure 2A). Tumor spheroid growth was quantified in a three-dimensional culture. As previously reported, control MCF10A-HER2 cells formed large acinar-like structures with a filled lumen [24]. Expression of VAPB in MCF10A-HER2 cells led to larger, irregular structures with invasive protrusions and a significant increase of spheroid size relative to parental and vector controls (Figure 2B). To determine whether increased tumor spheroid size is due to increased cell proliferation, the tumor spheroids were stained for PCNA, a cell proliferation marker. As shown in Figure 2C, VAPB expression elevated the number of PCNA-positive nuclei per spheroid, suggesting that VAPB regulates cell proliferation. Apoptosis, as measured by cleaved caspase 3 staining of 3D spheroid cultures, was not significantly changed (data not shown).

As an independent approach to determine whether VAPB is necessary for tumor cell growth, we knocked down VAPB in MMTV-Neu cells. These cells are derived from spontaneously arising mammary tumors from the MMTV-Neu mouse model [23]. Stable expression of two independent shRNAs significantly reduced VAPB protein levels (Fig. 3A) but not the related protein VAPA (Figure S1), suggesting that shRNAs specifically downregulated VAPB expression. In contrast to the morphology of vector control spheroids, which displayed disorganized and irregular structures with protrusions, knockdown of VAPB resulted in much smaller and more compact tumor spheroids. Since one of the VAPB knowndown (KD #1) targets the 3′ UTR, we were able to re-express full-length human VAPB protein (Figure 3B). KD cells with re-expression of VAPB restored tumor spheroid size and irregular morphology, demonstrating that phenotypes induced by shRNAs are VAPB specific and not due to off-target effects (Figure 3C). Consistent with the VAPB overexpression data, knockdown of VAPB significantly reduced proliferation in spheroid cultures as quantified by PCNA staining (Figure 3D). Together, these data indicate that VAPB regulates tumor spheroid size by increased tumor cell proliferation.

### VAPB Promotes Tumor Growth in an Orthotopic Mammary Tumor Model

Next, we investigated the role of VAPB in tumor growth *in vivo* in a mammary tumor orthotopic transplantation model [15]. One million MMTV-Neu control or VAPB knockdown cells were injected into cleared mammary gland fat pads of FVB recipient female mice. VAPB knockdown tumor cells failed to form tumors or formed very small, non-palpable tumors at five weeks post-transplantation, compared to parental or vector controls (Figure 4A). While parental or vector control tumors display densely packed tumor cells, VAPB knockdown tumors exhibit a reduced mammary tumor cell content (Figure 4B). To examine changes within the tumor epithelium, we assessed tumor proliferation and apoptosis in tissue sections by staining for Ki67 and cleaved caspase-3, respectively. We observed a significant decrease in tumor cells with Ki67 nuclear staining (Figure 4C), whereas cleaved caspase-3 levels were unaffected (data not shown). These data suggest that loss of VAPB inhibits HER2-initiated mammary tumor proliferation *in vivo*.

### VAPB-induced Cell Proliferation is Mediated by Elevated AKT Activity

To understand the mechanisms through which VAPB enhances proliferation, we investigated potential links between VAPB and signaling molecules relevant to tumor growth. Because ERK and AKT signaling pathways are two major players in regulating cell proliferation in mammary tumor cells, we assessed their activities by western blot analysis. MCF-10A.HER2 cells carrying wild-type VAPB expression construct or the control vector were serum-starved overnight and stimulated with EGF at the indicated time points. In response to EGF stimulation, there is a rapid increase in phosphorylation of AKT at Thr308 and Ser473, indicating activation of its kinase activity. The phospho-AKT levels were more pronounced in cells overexpressing VAPB than those carrying the control vector (Figure 5A). In contrast, ERK phosphorylation levels were not changed between two cell populations. Likewise, knockdown of VAPB in MMTV-Neu cells reduced phospho-AKT levels upon EGF treatment without affecting phospho-ERK. Re-expression of VAPB in KD cells restored AKT phosphorylation to control levels, indicating that AKT may play a key role in regulating cell growth in these cells. To test if VAPB-mediated spheroid growth is dependent on AKT phosphorylation, cells were treated with an allosteric AKT inhibitor [18]. AKT inhibition significantly reduced spheroid growth of VAPB expressing cells (Figure 5B and 5E). As expected the AKT inhibitor reduced phosphorylated AKT, while phospho-ERK was unaffected (Figure 5C and 5F). Although these results do not rule out the contribution of other signaling pathways, our findings suggest that VAPB-induced proliferation is mediated, at least in part, through activation of the AKT pathway.

## Discussion

VAPB was originally identified as one of the vesicle-associated membrane protein (VAMP) associated proteins. Although VAPB was implicated in a wide range of cellular processes, its function in cancer has not been characterized. In this report, we provide evidence that VAPB regulates mammary tumor growth and proliferation via activation of AKT activity. VAPB protein expression is elevated in primary and metastatic tumor specimens, and VAPB mRNA expression levels correlated negatively with patient survival in two large breast tumor datasets. Overexpression of VAPB increased spheroid size and proliferation in MCF10A-HER2 cells. Conversely, knockdown of VAPB in MMTV-Neu mammary tumor cells inhibited tumor cell proliferation in 3-D culture *in vitro* and suppressed tumor growth in orthotopic mammary tumor allografts. The growth regulation of mammary tumor cells controlled by VAPB appears to be mediated, at least in part, by modulation of AKT activities. Collectively, the genetic, functional and mechanistic analyses suggest a role of VAPB in tumor promotion in human breast cancer.

In breast cancer, approximately 30% of tumors have mutations in one or more components of the PI3K/AKT pathway [30]. Two lines of evidence suggest that VAPB expression modulates AKT activation. First, overexpression of VAPB in MCF10A-HER2 cells enhances phosphorylation of AKT, whereas knockdown of VAPB in MMTV-Neu tumor cells inhibited pAKT levels. Furthermore, the addition of an allosteric AKT inhibitor significantly reduced 3D spheroid growth induced by VAPB. It is currently unclear how VAPB regulates AKT activity. Since VAPB does not contain known enzymatic activities, it is likely that its action is mediated by its interaction with other proteins.

Major subcellular compartments where VAPB is localized are the ER and Golgi, where secretory and membrane proteins are synthesized and transported to cell surface [3], [4]. Several studies show that VAPB is required for neurotransmitter release and functions in early secretory transport events [5], [7]. Therefore it is possible that VAPB may regulate the protein secretion. Indeed, when VAPB is overexpressed in MCF10A-HER2 cells, transport of VSV-G-GFP ts045, a protein used to monitor vesicle traffic from ER/Golgi to the plasma membrane, is markedly enhanced (Figure S2), consistent with a previous report that show VAPB deficiency inhibited VSV-G-GFP ts045 to the plasma membrane in HeLa cells [31]. Accordingly, one mechanism by which VAPB promotes tumor cell proliferation could be through secretion of growth factors, cytokines, matrix metalloproteinases, as well as delivery of receptors to the cell surface [17]. This possibility is further strengthened by the fact that VAPB physically interacts with Arf1 and Rab1 (Figure S3), two small GTPases that are known to play critical roles in regulation of vesicle trafficking in the secretary pathway [32], [33], [34], [35], [36]. Interestingly, Arf1 has also been implicated in recruitment of p85 subunits of PI3K to EGFR in breast cancer cells [37], providing an additional possible mechanism by which VAPB could regulate AKT activities.

Aside from interacting with Arf1/Rab1, we and others also found VAPB in complex with other proteins including lipid transfer binding proteins such as Nir2 (Table S1) [22], [31], a phosphoinositol/phosphotidyalcholine transfer protein. Deletion of *scs2*, the yeast homolog of VAPs, was reported to reduce phosphoinosotide levels [38]. Since AKT phosphorylation is dependent on recruitment to the plasma membrane through interaction between the PH domain and PIP3, such alterations in phosphoinositols at the plasma membrane could affect ultimately the activation of AKT. Given the diverse function of VAPB-interacting proteins (Table S1), it is likely that multiple pathways converge through VAPB to enhance the AKT pathway and affect tumor cell proliferation.

Our results suggest that VAPB enhances breast tumor cell proliferation is mediated through the AKT pathway. However we cannot rule out other functions of VAPB that also may contribute to the phenotypes observed in this study, such as lipid sensing and transport [39], [40], [41] ER and Golgi architecture [22], [31], and the unfolded protein response [6], [10]. Therefore further studies are needed to address whether perturbation in these mechanisms alters the phenotypes of tumor cells in relation to VAPB expression. Furthermore, although full-length VAPB localizes to the ER, Golgi, and membrane-bound vesicles [3], [42], a number of studies showed that the MSP domain of VAPB can be secreted and act as an antagonist for Eph receptor tyrosine kinases in *C. elegans* and *Drosophila* [9], [43]. Secreted MSP has also been detected in human serum [9]. Because ligand-dependent EphA2 receptor signaling has been associated with tumor suppression whereas EphA2 ligand-independent signaling promotes tumor initiation and malignancy in breast cancer [44], [45], it is tempting to speculate that the secreted MSP domain may compete with ligand for binding to EphA2 receptor, thereby blocking EphA2-tumor suppressive function. However, although tumor cells do secrete MSP (Rao and Chen, unpublished data), further investigations are required to test this hypothesis.

In summary, we identified a functional role of VAPB in promoting tumor cell proliferation in breast cancer. This discovery opens up a number of exciting avenues for future studies of both full length VAPB and the secreted MSP domain in cancer. As VAPB overexpression is associated with poor patient survival, targeting VAPB-associated protein secretory pathway may provide novel targets for future pharmacological strategies in cancer therapy.

## Supporting Information

Figure S1 **Knockdown of VAPB does not affect VAPA expression.** VAPB expression was silenced in MMTV-Neu tumor cells by two independent lentiviral-mediated shRNAs (Figure 3A). VAPA protein expression was not affected by VAPB shRNA, as judged by western blot analysis, demonstrating the specificity of VAPB shRNAs. (Materials and Methods S1).
(EPS)

Figure S2 **VAPB expression does not affect apoptosis.** (A) MCF10A-HER2 or (B) MMTV-Neu knockdown cells were stained for cleaved caspase-3 at day 8 in 3-dimensional culture. Cleaved caspase-3 positive spheroids (green) were quantified by confocal microscopy analysis. No significant changes were observed in both cell lines. (C) in vivo analysis of apoptosis was measured by cleaved caspase-3 staining of tumor sections. Cleaved caspase-3 positive nuclei were quantified. VAPB deficiency does not significantly affect apoptosis in vivo. Arrows: cleaved caspase-3 positive cells. (Materials and Methods S1).
(EPS)

Figure S3 **PCNA analysis of tumor allografts.** Proliferating tumor cells were quantified by enumeration of PCNA- positive nuclei and presented as a percentage of PCNA+ nuclei/total nuclei. VAPB knockdown tumors showed a significant decrease in proliferation (p<0.05, ANOVA). Arrows: PCNA positive cells. (Materials and Methods S1).
(EPS)

Figure S4 **Analysis of AKT and ERK activities in VAPB expressing cells.** MCF10A-HER2 (A) and MMTV-Neu knockdown cells (B) expressing VAPB or carrying control vector were serum starved and stimulated with 20 ng/mL EGF at the indicated time points. Phospho-ERK levels were measured by western blot analysis and quantified. (C) VAPB was knocked down in MMTV-Neu cells (KD#1) and re-expressed via retroviral transduction (KD#1_VAPB). Phospho-AKT and phospho-ERK levels were measured by western blot analysis. Representative blots from 3 independent experiments are shown. (Materials and Methods S1).
(EPS)

Figure S5 **PI3K inhibition attenuates VAPB dependent spheroid growth.** (A) Cells were cultured in 3D Matrigel for 2 days and then treated with LY294002 (20 µM) or vehicle control every 2 days. Tumor cell spheroids were quantified at day 8. (B) Inhibition of AKT activity was confirmed by western blot analysis for phospho-AKT levels. Shown were representative blots from two independent experiments. (Materials and Methods S1).
(EPS)

Figure S6 **VAPB facilitates the transport of secretory proteins to cell surface.** (A) MCF10A-HER2-VAPB expressing cells were transfected with the ts045 temperature sensitive vesicular stomatitis viral glycoprotein (VSVG) GFP and incubated at 40°C for 16 hours to accumulate misfolded VSVG protein in the ER. Following a 30-minute incubation with cyclohexamide, the cells were shifted to a permissive temperature (32°C) to allow transport along the secretory pathway. Total VSVG was visualized by GFP fluorescence (green) and cell surface VSVG was detected using an antibody against VSVG ectodomain (red) under non-permeable condition. (B) The kinetics of appearance of VSVG-GFP at the cell surface was measured by cell-surface biotinylation and subsequent quantification of immnoblots with anti-VSVG in two independent experiments (p<0.05, unpaired t test). (Materials and Methods S1).
(EPS)

Figure S7 **VAPB interacts with Arf1 and Rab1 small GTPases.** HEK 293T cells were co-transfected with VAPB-Myc and Arf1-HA or FLAG-Rab1 expression constructs. VAPB and Arf1 or Rab1 was immunoprecipitated from cell lysates by anti-Myc, anti- HA, or anti-FLAG, and the resulting protein complexes were analyzed by western blot for Arf1 (A) and Rab1b (B) or VAPB, respectively. (Materials and Methods S1).
(EPS)

Table S1 **Representative Candidates of VAPB binding proteins.** MMTV-Neu control or VAPB knockdown cells were treated with chemical crosslinkers prior to lysis. Cell lysates were immunoprecipitated with anti-VAPB and resulting protein complexes were subjected to mass spectrometry analysis. The following criteria were used for selection of candidate proteins: (1) spectral counts ≥5 and (2) the ratio of vector/knockdown ≥4. 170 candidate proteins were classified based on biological processes as annotated in PANTHER [5]. Selected functional groups and proteins are listed in TableS1.
(EPS)

Materials and Methods S1. (DOC)
