# Supplementary material for: VAMP-Associated Protein B (VAPB) Promotes Breast Tumor Growth by Modulation of Akt Activity
Source: PLoS One. 2012 Oct 1;7(10):e46281. doi: 10.1371/journal.pone.0046281 (PMC3462209; doi:10.1371/journal.pone.0046281)
Supplement: Materials and Methods S1. — (DOC) [file pone.0046281.s009.doc]

**Materials and Methods S1**

**Antibodies, reagents and plasmids**

Antibodies against the following proteins were used: anti-VAPA (K-14), anti-actin (I-19), anti-Arf1 (Arfs1A9/5), and agarose-congugated anti-HA for immunoprecipitation studies (Santa Cruz Biotechnologies), anti-HA (Sigma), anti-MYC, anti-cleaved caspase-3 (Cell Signaling Technologies) and anti-VSVG (8G5F11) (KeraFast). C-terminus tagged VAPB-Myc or Arf1-HA and FLAG-Rab1b was subcloned into pCLSXN_Neo (Addgene) or pCDNA3. 1 (Invitrogen) using cDNAs obtained from the Vanderbilt Microarray Core. Growth factor-reduced Matrigel were purchased from BD Biosciences. Cell permeable crosslinkers (DSP and DTME) were purchased from Thermo Scientific. For PI3K inhibition, LY204002 was purchased from Sigma. PCNA staining kit was purchased from Invitrogen.

**Identification of VAPB-interacting proteins**

Proteins in complex with VAPB were identified using ReCLIP (Reversible Cross-Link Immuno-Precipitaiton) technology as previously described [1]. Briefly, to preserve physiological complexes, MMTV-Neu Vector or VAPB knockdown cells (KD #1) were treated with membrane permeable cross-linkers (DSP and DTME) prior to lysis. DSP and DTME were first dissolved in DMSO (20mM) then mixed in a 1:1 ratio, with a final concentration of 0.4mM in PBS. Cells were washed twice with PBS and incubated with the cross-linker mixture for 30 minutes at room temperature. The reaction was quenched for 10 minutes and cells were subsequently lysed and subjected to coimmunoprecipitation with anti-VAPB conjugated Protein G Dynabeads (Invitrogen). VAPB and its binding partners were eluted by incubating the beads at 37°C for 30 minutes in the presence of 100mM DTT. For mass spectrometry analysis, the eluates were boiled for 5 minutes in freshly prepared Laemmli sample buffer. Eluted and boiled proteins from MMTV-Neu vector and VAPB knockdown samples were trichloroacetic acid -precipitated and processed for shotgun analysis using Multidimentional Protein Identification Technology (MudPIT). Mass spectra data was analyzed using IDPICKER [2] software, an open-source protein assembly tool that derives a minimum protein list from peptide identifications filtered to a specified False Discovery Rate of less that 5%. This service was provided by the Mass Spectrometry Research Center at Vanderbilt University.

**VSVG-GFP trafficking assays**

MCF10A-HER2 cells were transfected with VSV-G-GFP ts045 (generous gift from Dr. Jennifer Lippencott-Swartz) using Lipofectamine 2000. After 3 hours, transfection media was replaced with growth media and immediately incubated at 40°C for an additional 16 hours. Cells were then treated with 100ug/mL cyclohexamide (Sigma-Aldrich) for 30 minutes prior to shift to 32°C at the indicated time points. For immunofluorescence analysis of VSVG-GFP, cells were plated on glass cover slips were rinsed in PBS, then fixed without permeabilization in 2% PFA for 15 minutes at room temperature and probed with ectodomain- specific anti-VSVG (1:1000). For biochemical analysis of surface VSVG-GFP, cells were labeled with Sulfo- NHS-LC-Biotin (Thermo Scientific) and processed as previously described [3]. Surface labeled proteins were pulled down using Streptavidin-agarose resin (Thermo Scientific) and subjected to western blot analysis. Cell surface and total VSVG protein was probed with monoclonal anti-VSVG (1:1000) and detected with anti-mouse HRP. Densitometry analysis for band intensity was performed using NIH Image J software. Cell surface VSVG was calculated as a percent of total VSVG. Data are derived from two independent experiments.

**Immunoblot and co-immunoprecipitation**

For immunoblot analysis of VAPA, cells were washed with PBS and lysed in Radioimmunoprecipitation Assay (RIPA) buffer (50mM Tris pH 7.4, 150mM NaCl, 1% NP-40, 0.5% deoxycholic acid, 0.1% SDS) supplemented with protease and phosphatase inhibitor tablets (Roche). Approximately 30-50ug of total cell lysate was separated by SDS-PAGE and probed with anti-VAPB (1:1000) overnight in 5% nonfat dry milk/ TBSTween (0.05%) and detected with anti-goat HRP (1:1000). VAPB and Arf1 coimmunoprecipitation in HEK 293T cells was performed with the addition of 2mM DSP (dithiobis succinimidyl propionate), a reversible chemical cross linker and processed as previously described [4].

**References**

1. Smith AL, Friedman DB, Yu H, Carnahan RH, Reynolds AB (2011) ReCLIP (reversible cross-link immuno-precipitation): an efficient method for interrogation of labile protein complexes. PLoS One 6: e16206.

2. Ma ZQ, Dasari S, Chambers MC, Litton MD, Sobecki SM, et al. (2009) IDPicker 2.0: Improved protein assembly with high discrimination peptide identification filtering. J Proteome Res 8: 3872-3881.

3. Ying M, Grimmer S, Iversen TG, Van Deurs B, Sandvig K (2003) Cholesterol loading induces a block in the exit of VSVG from the TGN. Traffic 4: 772-784.

4. Amarilio R, Ramachandran S, Sabanay H, Lev S (2005) Differential regulation of endoplasmic reticulum structure through VAP-Nir protein interaction. J Biol Chem 280: 5934-5944.

5. Thomas PD, Campbell MJ, Kejariwal A, Mi H, Karlak B, et al. (2003) PANTHER: a library of protein families and subfamilies indexed by function. Genome Res 13: 2129-2141.
